# Anderson v. Moeller

**Published:** 1900-05-19

**Authors:** 


### ANDERSON V. MOELLER.

The case of Anderson and another v. Moeller, which occupied the Lord Chief Justice of England and a jury during two days of last week, is full of points of interest for the medical profession. The plaintiffs, Dr. W. M. A. Anderson, of 37, Wimpole Street, and Dr. Fenner, of Birchin Lane, who were in partnership, sued the defendant for the purpose of recovering their charges, amounting to £144 18s., for medical and surgical attendance upon him. It appeared that the defendant was a stockbroker's clerk, and that he went to Dr. Fenner on April 5th, 1899, in order to consult him as to a swollen ankle and other symptoms from which he was suffering. Dr. Fenner advised him to go into a nursing home in Devonshire Street, and he did so, and was attended there mainly by Dr. Anderson, presumably because that gentleman lived near at hand. The illness continued, and the original swelling of the ankle became complicated by phlebitis, and subsequently by contraction of the leg. Both Mr. Alfred Cooper and Sir Lauder Brunton were consulted in the course of the case, and Sir Lauder paid three visits. The patient would appear to have been difficult to deal with from the very first, and on more than one occasion to have resisted the application of treatment. He was greatly alarmed by the sight of a cylinder which was brought for the purpose of administering a hot-air bath, and is said to have struggled and shouted when an endeavour was made to apply it. In May Dr. Anderson straightened the affected leg under the influence of an anæsthetic, to the administration of which the patient strongly objected, and in June, when it was found necessary to repeat the operation, he refused the anæsthetic altogether, and the straightening was effected without it, in circumstances as to which there was some conflict of evidence. On June 28th Dr. Anderson ordered a soothing draught, and, when he called on the following day, found that the patient had taken also a dose of antipyrine. Dr. Anderson objected to this, and expressed his desire to withdraw from the case, a course to which the patient assented. He then refused to pay Dr. Anderson for his attendance, and raised the defence that the services rendered had been of no value or benefit to him by reason of the plaintiff's negligence and want of professional skill, and he counterclaimed for damages on account of the alleged negligence. The jury returned a verdict for the plaintiffs for 100 guineas, and found for them on the counterclaim. The alleged negligence and want of skill were therefore disproved; and, in point of fact, no serious endeavour was made by the defence to sustain them.

We must, of course, congratulate Dr. Anderson on that part of the verdict which covers the allegation of negligence or unskilfulness; but, looking at the case as a whole, we must confess that it does not incline us to think more favourably than before of legal proceedings for the recovery of medical charges. There is no doubt, of course, that a doctor is as much entitled to recover his charges as the member of any other calling; but the expediency of adopting such a course may not always be so certain. In this particular instance, for example, it cannot be supposed that Dr. Anderson has gained anything. It is hardly likely that 100 guineas will cover his own costs as between attorney and client, to say nothing of recompense for the worry, anxiety, and vexation which he must have experienced. It must have been perfectly evident to Dr. Anderson, from a very early stage of the attendance, that the characteristics and peculiarities of Mr. Moeller were such as hardly to give any prospect of fair play to his medical advisers, with whom he was clearly not inclined to co-operate for his own good; and, in such circumstances, we cannot but think it might have been more discreet to have retired from the case at an earlier period, and to have left so intractable a subject to his own devices. There is, we consider, an unwritten compact between patient and doctor that each should do his best towards the attainment of the object which both alike should have in view. Where this compact is not recognised, or is not observed, the sooner the doctor retires from an untenable position the better it will be, speaking generally, alike for his pocket, his reputation, and his comfort; and, where it is recognised and observed, he will be able to retain a position which is, we think, more dignified than that of a plaintiff, and to range himself side by side with Chaucer's "Persoun," of whom it is recorded—

"Ful loth were him to curse for his tythes."

